# The Relationship between Retinol-Binding Protein 4 and Markers of Inflammation and Thrombogenesis in Children with Kawasaki Disease

**DOI:** 10.1155/2021/7029514

**Published:** 2021-01-11

**Authors:** Maoling Yang, Haobo Weng, Qiongfei Pei, Fengchuan Jing, Qijian Yi

**Affiliations:** ^1^Department of Cardiovascular Medicine, National Clinical Research Center for Child Health and Disorders, Ministry of Education Key Laboratory of Child Development and Disorders, China International Science and Technology Cooperation Base of Child Development and Critical Disorders, Children's Hospital of Chongqing Medical University, Chongqing, China; ^2^Chongqing Key Laboratory of Pediatrics, Chongqing 400014, China

## Abstract

**Background:**

Kawasaki disease (KD) is a self-limited vasculitis with unknown etiologies, and coronary artery lesions (CALs) are the most common and serious complications. Retinol-binding protein 4 (RBP4) has been confirmed effects on vasodilation, platelet activation inhibition, and cardiovascular diseases by researches. Therefore, this study was aimed at investigating the relationship between RBP4 and inflammation as well as thrombogenesis in children with KD.

**Methods:**

79 subjects were from 62 children with KD and 17 healthy controls (HCs). The KD group was divided into KD with CALs (KD-CALs) and KD without CALs (KD-NCALs), and the serum RBP4 levels were measured by enzyme-linked immunosorbent assay (ELISA).

**Results:**

Compared with the HC group, serum RBP4 levels in the KD group were significantly decreased (*p* < 0.05). RBP4, hemoglobin (Hb), and mean platelet volume (MPV) levels were higher, while platelet counts (Plt) and thrombin time (TT) levels were lower in the KD-NCALs group than in the KD-CALs group (*p* < 0.05). RBP4 had positive correlation with time point of intravenous immunoglobulin (IVIG), Hb, and percentage of leukomonocytes (L%) and negative correlation with the percentage of neutrophils (N%), MPV, C-reactive protein (CRP), neutrophil-to-lymphocyte ratio (NLR), prothrombin time (PT), fibrinogen (Fbg), and D-dimer (DD) in the KD group; RBP4 had positive correlation with the time point of IVIG and L% and negative correlation with N%, MPV, and NLR in the KD-NCALs group; and RBP4 had positive correlation with Hb and L% and negative correlation with N%, CRP, NLR, and PT in the KD-CALs group (*p* < 0.05). Multiple linear regression analysis confirmed that Hb and CRP in the KD group, MPV and N% in the KD-NCALs group, and PT and CRP in the KD-CALs group were independent predictors of RBP4 (*p* < 0.05).

**Conclusion:**

Lower RBP4 was observed in the KD group than in the HC group, and RBP4 had associations with markers of inflammation and thrombogenesis in children with KD.

## 1. Introduction

Kawasaki disease (KD) is a vasculitis of small and medium vessels and mainly affects young children under 3 years old, and the exact etiology of KD is still unclear. Coronary artery lesions (CALs), thrombogenesis [1, 2], and even myocardium infarction are the most serious complications in children with KD, and so KD has been taken as the main cause of children with acquired heart diseases now. It has been reported that inflammatory cytokines and oxidative stress will insult in endothelial dysfunction of coronary artery in acute KD [3]. Our previous studies have showed that serum cytokines, such as adiponectin, resistin, C1q/tumor necrosis factor-related protein-1 (CTRP1), tumor necrosis factor-*α* (TNF-*α*), interleukin-1*β* (IL-1*β*), and interleukin-6 (IL-6), were involved in the process of CALs in KD [4–6].

Retinol-binding protein 4 (RBP4), a newly discovered adipokine, is the main transport protein of retinol (vitamin A) [7] and is mainly secreted by adipocytes and hepatocytes [8]. Evidences show that RBP4 acts as a metabolic marker of chronic inflammatory diseases such as obesity, type 2 diabetes, and cardiovascular disease (CVD) [9–11] and has relation with the severity and prognosis of acute ischemia stroke (AIS), stable coronary artery disease (CAD), acute coronary syndrome (ACS), and atherosclerosis [12–19]. However, there is still no study on the relationship between RBP4 and KD. Therefore, the aim of our study was to investigate the relationship between RBP4 and inflammation as well as thrombogenesis in children with KD.

## 2. Methods

### 2.1. Subjects

62 KD patients as the KD group, 42 males and 20 females, average age 2.51 ± 0.99 years old, and 17 healthy children as the healthy control group (HC), 10 males and 7 females, average age 2.69 ± 0.70 years old, were enrolled in a consecutive manner from the Children's Hospital of Chongqing Medical University between November 2018 and August 2019. The present study was a cross-sectional design study. All of the KD patients were diagnosed in strict accordance with the criteria proposed by the Japanese Circulation Society Joint Working Group [20], and those who have metabolic diseases or immunological diseases were excluded.

Professional pediatricians collected baseline information including age, gender, growth and development history, medical history, and family history and performed comprehensive physical examinations for all subjects. Body weight and height were measured according to a uniform standard in all subjects. Echocardiography was performed by one experienced ultrasound expert on the KD patients within 2 weeks of the onset and before administration of intravenous immunoglobulin (IVIG) and anticoagulants. The echocardiographic diagnosis based on the calculation standard of *Z* score and CALs evaluation standard is as follows [21, 22]: no dilation of coronary artery, *Z* score less than 2.5; small coronary artery aneurysm (CAA) or coronary artery dilation, *Z* score between 2.5 and 5; large CAA, *Z* score between 5 and 10; and giant CAA, *Z* score equal to or greater than 10. CALs included CAA or coronary artery dilation, large CAA, and giant CAA. According to inner diameter of coronary by echocardiography and the calculation standard of the *Z* score, the 62 KD patients were divided into two groups: KD without CALs (KD-NCALs; *n* = 24) and KD with CALs (KD-CALs; *n* = 38). Blood samples were collected in tubes containing liquid EDTA for complete blood count (CBC), C-reactive protein (CRP), and erythrocyte sedimentation rate (ESR) determination, in a tube with a clot activator (and gel separator) for procalcitonin (Pct), aspartate aminotransferase (AST), alanine aminotransferase (ALT), and creatine kinase-MB (CK-MB) determination, and in a tube with sodium citrate for prothrombin time (PT), activated partial thromboplastin time (APTT), fibrinogen (Fbg), thrombin time (TT), and D-dimer (DD) determination (coagulation test). White blood cell counts (WBC), platelet counts (Plt), hemoglobin (Hb), percentage of neutrophils (N%), percentage of leukomonocytes (L%), mean platelet volume (MPV), and platelet distribution width (PDW) were assessed by a Sysmex XE-2100 hematology analyzer (Sysmex, Japan); CRP was assessed by a gold standard digital quantitative analyzer (UPPER, China); ESR was assessed by a VISION automatic dynamic erythrocyte sedimentation rate analyzer (YHLO, China); Pct was assessed by a Roche Cobas 8000 automatic electrochemiluminescence immunoassay analyzer (Roche, Germany); AST, ALT, and CK-MB were assessed by a Vitros-350 automatic dry biochemical analyzer (Johnson& Johnson, USA); and PT, APTT, Fbg, TT, and DD were assessed by a Sysmex CS 5100 automatic blood coagulation analyzer (Sysmex, Japan). Blood samples were drawn before administration of IVIG and anticoagulants.

Blood samples were collected from KD patients (before IVIG) and HC patients and then centrifuged at 3000 rpm/min for 10 minutes to separate the serum immediately. Serum samples were stored at -80°C. The concentration of serum RBP4 was detected by a sandwich enzyme-linked immunosorbent assay (ELISA) kit (R&D, USA).

### 2.2. Calculations

Neutrophil-to-lymphocyte ratio (NLR) and platelet-to-lymphocyte ratio (PLR) were calculated as neutrophil and platelet counts divided by lymphocyte counts, respectively. Body mass index (BMI) was calculated by weight in kilograms dividing height in meters squared.

Informed consent was obtained from the legal guardian of each patient. This research was approved by the ethics committee of the Children's Hospital of Chongqing Medical University. The study protocol conforms to the ethical guidelines of the 1975 Declaration of Helsinki as reflected in a priori approval by the institution's human research committee.

### 2.3. Statistical Analysis

SPSS version 19 (IBM Corp, Armonk, NY, USA) was used for analysis of the clinical data and laboratory variables. Normality of data was checked using the Shapiro-Wilk test. The measurement data conformed to a normal distribution and was expressed as means with standard deviations. The measurement data was consistent with the skewness distribution and described by the median (interquartile range). Independent sample *T*-test and applicable *χ*^2^ test were used to compare the baseline data. Mann-Whitney *U* test was used for statistical analysis of continuous data without normal distribution, and an independent sample *T*-test was used for statistical analysis of continuous data with normal distribution. Spearman's rho test was used to determine the correlations with the data of nonnormal distribution. Pearson's correlation test was used for normal distribution data. CRP and NLR were logarithmically transformed into normal distribution for Pearson's correlation analysis and multiple linear regression analysis. For multiple linear regression analysis, variables entered into the stepwise forward model were normally distributed, showed significant parametric correlations with RBP4, and showed no multicollinearity. RBP4 was a dependent variable of correlation analysis and multiple linear regression analysis. *p* < 0.05 was considered statistically significant.

## 3. Results

### 3.1. The Levels of Serum RBP4 in KD Group and HC Group

There were no significant differences in the sex ratio (*χ*^2^ = 0.472, *p* = 0.492) and age between the two groups (*p* > 0.05).

**Figure 1:**
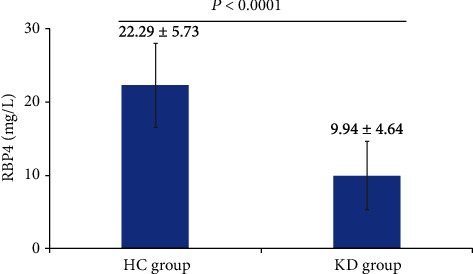
The concentration of serum RBP4 in two groups.

Compared with the HC group, the levels of serum RBP4 were significantly decreased in the KD group (*p* < 0.05) (Figure 1).

### 3.2. Clinical and Laboratory Indexes and Serum RBP4 Levels in KD-NCALs and KD-CALs Groups

The levels of RBP4, Hb, and MPV were higher in the KD-NCALs group than in the KD-CALs group (*p* < 0.05). Moreover, the levels of Plt and TT were significantly lower in the KD-NCALs group than in the KD-CALs group (*p* < 0.05). However, there were no significant differences in age, gender, the time point of IVIG, WBC, N%, L%, PDW, CRP, ESR, Pct, AST, ALT, NLR, PLR, CK-MB, PT, APTT, Fbg, DD, and BMI between the two groups (*p* > 0.05) (Table 1).

### 3.3. Correlations between RBP4 and Clinical and Laboratory Indexes

RBP4 showed no correlation with WBC, Plt, PDW, ESR, Pct, AST, CK-MB, PLR, APTT, TT, and BMI (*p* > 0.05); positive correlation with the time point of IVIG, Hb, and L% (*p* < 0.05); and negative correlation with N%, MPV, CRP, ALT, NLR, PT, Fbg, and DD (*p* < 0.05) in KD patients (Table 2).

Serum RBP4 had positive correlation with the time point of IVIG and L%; negative correlation with N%, MPV, and NLR in the KD-NCALs group (*p* < 0.05); and no significant correlation with WBC, Plt, Hb, PDW, CRP, ESR, Pct, AST, ALT, CK-MB, PLR, PT, APTT, Fbg, TT, DD, and BMI (*p* > 0.05). Serum RBP4 showed no significant correlation with the time point of IVIG, WBC, Plt, MPV, PDW, ESR, Pct, AST, CK-MB, PLR, APTT, Fbg, TT, DD, and BMI in the KD-CALs group (*p* > 0.05), positive correlation with Hb and L%, and negative correlation with N%, CRP, ALT, NLR, and PT in the KD-CALs group (*p* < 0.05) (Table 2).

Using the time point of IVIG, Hb, N%, L%, MPV, CRP, NLR, PT, Fbg, and DD as independent variables, the stepwise multiple linear regression analysis indicated that Hb (standardized coefficient beta = 0.481, *p* < 0.001) and CRP (standardized coefficient beta = −0.330, *p* = 0.009) in the KD group; using the time point of IVIG, N%, L%, MPV, and NLR as independent variables, the stepwise multiple linear regression analysis indicated that MPV (standardized coefficient beta = −0.526, *p* = 0.003) and N% (standardized coefficients beta = −0.383, *p* = 0.025) in the KD-NCALs group; and using Hb, N%, L%, CRP, NLR, and PT as independent variables, the stepwise multiple linear regression analysis indicated that PT (standardized coefficient beta = −0.391, *p* = 0.024) and CRP (standardized coefficient beta = −0.352, *p* = 0.040) in the KD-CALs group were independent predictors of RBP4 (Table 3).

## 4. Discussion

In the present study, we found that the concentrations of serum RBP4 in children with KD were lower than those in the HC group, which may be the effect of inflammation and acute-phase response on retinol binding protein (RBP) [23]. Moreover, studies have shown that RBP4 induced the expression of NO synthase (NOS) subunit with anti-inflammatory and vasodilatory effects by stimulating phosphatidylinositol 3-kinase (PI3K)/protein kinase B (Akt)/NOS pathway in human endothelial cells and promoted the release of vasodilatory eicosanoid PGI2 in smooth muscle cells, a prostaglandin, with the effect of vasodilation and platelet activation inhibition. Overall, these results indicate that RBP4 acts as a beneficial role in vascular function [24–26]. There studies have shown that a high level of serum RBP4 may be associated with a higher cardiovascular risk in overweight/obese adolescent girls [27], a high level of baseline RBP4 in childhood was associated with an adverse cardiovascular risk profile upon a 10-year follow-up study [28], and the relationship between RBP4 and cardiovascular sequelae of obesity children appears to be secondary to the underlying association with body fat [29]. On the other hand, in a study on children with inflammatory bowel disease, RBP4 had a negative correlation with disease activity, which is conducive to the intervention of inflammation in disease development [30].

CRP, an inflammatory biomarker, has an association with KD and CALs [4], and our study showed that there was a significant negative correlation between RBP4 and CRP in the KD group and the KD-CALs group, so we have reason to speculate that RBP4 should be involved in inflammatory pathogenesis of KD and further has effect on CALs. In recent years, PLR and NLR have become a new parameter of inflammation and play a central role in the inflammation pathogenesis in KD and CALs [31, 32]. There was negative correlation between RBP4 and NLR in children with KD, which suggested that there is a negative correlation between RBP4 with inflammatory response of KD. In other words, RBP4, as an adipocytokine, may have a negative regulation effect on the uncontrolled inflammatory response in KD. In many inflammation-related diseases such as acute disease, operation, or trauma, lower levels of serum RBP4 were observed [33]. Therefore, RBP4 can be used as not only a nutritional evaluation protein but also an antiacute phase protein [30]. Researchers have reported that N% and L% are indexes of inflammation in KD [5]. The results showed that the level of RBP4 was positively correlated with L% and negatively correlated with N% in KD patients, further documenting RBP4 involvement in the inflammatory response of KD.

Endothelial cell inflammation and injury as well as a high level of platelet in KD patients result in hypercoagulability and thrombosis in the acute phase and are prone to myocardium infarction [34, 35]. In our study, RBP4 was negatively correlated with MPV, PT, Fbg, and DD in KD patients, negatively correlated with MPV in the KD-NCALs group, and negatively correlated with PT in the KD-CALs group, which suggested that RBP4 may be a potential protective factor on coagulation function and thrombosis in KD. And previous studies have shown that RBP4 can regulate vasodilation and inhibit platelet activity to protect vascular function via inducing the expression of NO synthase (NOS) subunit and promoting the release of vasodilatory eicosanoid PGI2, which is consistent with our results [24–26]. Moreover, we found that Hb and CRP in KD patients, MPV and N% in KD patients without CALs, and PT and CRP in KD patients with CALs were independent predictors of RBP4, which may indicate that the serum levels of RBP4 were influenced by these parameters, which further affected the development of CALs in KD.

Some limitations of this study warrant discussions. Due to the limited number of cases, the sample size was small, and expanding the observation of the sample size could help us to confirm the causal relationship between RBP4 and coronary artery lesions in KD patients. Second, the study that was cross-sectional could limit us to explore the association between RBP4 and coronary artery lesions in KD patients. Finally, the measurement of RBP4 levels was done by ELISA rather than by quantitative western blotting, which are standardized to the full-length RBP4 protein. However, the results of this study to some extent supported the hypothesis that RBP4 may have associations with KD and CALs.

## 5. Conclusions

In summary, the levels of serum RBP4 were significantly lower in children with KD than in healthy controls, and the serum RBP4 levels were even lower in KD patients with CALs than in KD patients without CALs in our cross-sectional study. Meanwhile, RBP4 levels were negatively correlated with inflammatory indicators and coagulation indexes and positively correlated with Hb. Moreover, Hb and CRP in KD patients, MPV and N% in KD patients without CALs, and PT and CRP in KD patients with CALs were independent predictors of RBP4. Based on these findings, we can conclude that there may be a potential link between RBP4 and the development of CALs in KD, but the mechanism needs further study.

## Figures and Tables

**Table 1:** Basic characteristics of all KD patients.

	KD-NCALs	KD-CALs	*p*
*n*	24	38	
Age (years)	2.88 ± 0.99	2.32 ± 0.96	0.147
Gender (male/female)	16/8	26/12	0.886
Time point of IVIG (days)	5.9 ± 1.2	5.8 ± 1.6	0.905
WBC (10^3^/*μ*L)	12.7 ± 1.8	13.4 ± 3.6	0.558
Plt (10^3^/*μ*L)	363.1 ± 80.2	434.4 ± 76.3	0.010^∗^
Hb (g/L)	109.9 ± 7.8	103.8 ± 8.8	0.008^∗^
N% (%)	0.6 ± 0.2	0.5 ± 0.2	0.066
L% (%)	0.3 ± 0.2	0.4 ± 0.2	0.149
MPV (fL)	10.5 ± 1.4	9.7 ± 0.7	0.003^∗^
PDW (fL)	11.9 ± 0.8	12.0 ± 1.0	0.826
CRP (mg/dL)	40 (32-75)	45 (24-60)	0.408
ESR (mm/hour)	61 (47-84)	70 (58-84)	0.317
Pct (ng/mL)	0.4 (0.2-0.8)	0.5 (0.2-1.1)	0.895
AST (U/L)	26 (22-38)	26 (21-33)	0.778
ALT (U/L)	22 (16-43)	23 (15-57)	0.515
NLR (10^3^/*μ*L/10^3^/*μ*L)	2.1 (1.6-3.6)	1.6 (0.7-10.2)	0.120
PLR (10^3^/*μ*L/10^3^/*μ*L)	131.0 ± 19.8	132.8 ± 17.6	0.841
CK-MB (U/L)	0.9 ± 0.3	0.9 ± 0.4	0.703
PT (s)	12.0 ± 0.9	11.9 ± 0.9	0.688
APTT (s)	29.5 ± 2.0	27.2 ± 2.1	0.321
Fbg (g/L)	5.9 ± 1.1	6.0 ± 1.6	0.814
TT (s)	15.5 ± 1.4	16.7 ± 2.5	0.048^∗^
DD (mg/L)	1.2 ± 0.6	1.3 ± 0.6	0.867
RBP4 (mg/L)	11.5 ± 5.9	8.8 ± 3.2	0.020^∗^
BMI (kg/m^2^)	16.1 ± 1.9	16.7 ± 2.1	0.358

Abbreviations: IVIG: immunoglobulin intravenous; WBC: white blood cell counts; Plt: platelet counts; Hb: hemoglobin; N%: percentage of neutrophils; L%: percentage of leukomonocytes; MPV: mean platelet volume; PDW: platelet distribution width; CRP: C-reactive protein; ESR: erythrocyte sedimentation rate; Pct: procalcitonin; AST: aspartate aminotransferase; ALT: alanine aminotransferase; CK-MB: creatine kinase-MB; PT: prothrombin time; APTT: activated partial thromboplastin time; Fbg: fibrinogen; TT: thrombin time; DD: D-dimer; NLR: neutrophil-to-lymphocyte ratio; PLR: platelet-to-lymphocyte ratio; BMI: body mass index. ^∗^*p* < 0.05; values represent mean ± SD and median (interquartile range).

**Table 2:** Correlations of RBP4 with clinical and laboratory variables in KD patients.

	KD (*n* = 62)		KD-NCALs (*n* = 24)		KD-CALs (*n* = 38)	
	*r*	*p*	*r*	*p*	*r*	*p*
Time point of IVIG (day)	0.287	0.028^∗^	0.624	0.001^∗^	0.041	0.815
WBC (10^3^/*μ*L)	-0.136	0.445	-0.025	0.934	-0.273	0.231
Plt (10^3^/*μ*L)	-0.123	0.469	0.200	0.475	-0.102	0.652
Hb (g/L)	0.378	0.002^∗^	0.303	0.150	0.359	0.027^∗^
N% (%)	-0.299	0.018^∗^	-0.465	0.022^∗^	-0.408	0.011^∗^
L% (%)	0.327	0.009^∗^	0.489	0.015^∗^	0.408	0.011^∗^
MPV (fL)	-0.279	0.028^∗^	-0.585	0.003^∗^	-0.111	0.509
PDW (fL)	0.063	0.749	-0.220	0.601	0.183	0.439
CRP (mg/dL)^#^	-0.329	0.016^∗^	-0.262	0.251	-0.547	0.001^∗^
ESR (mm/hour)	-0.018	0.900	-0.169	0.453	0.113	0.552
Pct (ng/mL)	-0.146	0.269	0.001	0.995	-0.273	0.107
AST (U/L)	0.009	0.946	-0.117	0.604	0.108	0.524
ALT (U/L)	-0.305	0.019^∗^	0.002	0.994	-0.445	0.006^∗^
CK-MB (U/L)	-0.065	0.675	-0.191	0.462	0.120	0.550
NLR (10^3^/*μ*L/10^3^/*μ*L)^#^	-0.311	0.014^∗^	-0.466	0.022^∗^	-0.405	0.012^∗^
PLR (10^3^/*μ*L/10^3^/*μ*L)	-0.158	0.506	-0.096	0.821	-0.203	0.527
PT (s)	-0.393	0.002^∗^	-0.385	0.070	-0.517	0.001^∗^
APTT (s)	-0.071	0.608	-0.109	0.620	0.058	0.757
Fbg (g/L)	-0.272	0.034^∗^	-0.303	0.159	-0.319	0.051
TT (s)	0.095	0.467	0.198	0.366	0.219	0.186
DD (mg/L)	-0.280	0.035^∗^	-0.296	0.171	-0.328	0.059
BMI (kg/m^2^)	0.009	0.957	0.093	0.713	0.020	0.927

Abbreviations: IVIG: immunoglobulin intravenous; WBC: white blood cell counts; Plt: platelet counts; Hb: hemoglobin; N%: percentage of neutrophils; L%: percentage of leukomonocytes; MPV: mean platelet volume; PDW: platelet distribution width; CRP: C-reactive protein; ESR: erythrocyte sedimentation rate; Pct: procalcitonin; AST: aspartate aminotransferase; ALT: alanine aminotransferase; CK-MB: creatine kinase-MB; PT: prothrombin time; APTT: activated partial thromboplastin time; Fbg: fibrinogen; TT: thrombin time; DD: D-dimer; NLR: neutrophil-to-lymphocyte ratio; PLR: platelet-to-lymphocyte ratio; BMI: body mass index. ^#^Logarithmic transformation of non-normal distribution. ^∗^*p* < 0.05.

**Table 3:** Significant predictors of RBP4 using stepwise linear regression.

Coefficients					
Group	Unstandardized coefficients		Standardized coefficients	*t*	*p*
	*B*	Std. error	Beta		
KD group					
Hb	0.198	0.050	0.481	3.957	<0.001
CRP	-3.560	1.311	-0.330	-2.716	0.009
KD-NCALs					
MPV	-2.232	0.672	-0.526	-3.321	0.003
N%	-13.551	5.598	-0.383	-2.421	0.025
KD-CALs					
PT	-1.009	0.423	-0.391	-2.385	0.024
CRP	-3.061	1.426	-0.352	-2.147	0.040

Abbreviations: Hb: hemoglobin; CRP: C-reactive protein; MPV: mean platelet volume; N%: percentage of neutrophils; PT: prothrombin time.

## Data Availability

The clinical and laboratory data of all human subjects used to support the findings of this study are included within the article.
